# Supplementary material for: Evidence for close molecular proximity between reverting and undifferentiated cells
Source: BMC Biol. 2022 Jul 6;20:155. doi: 10.1186/s12915-022-01363-7 (PMC9258043; doi:10.1186/s12915-022-01363-7)
Supplement: Supplementary file 1 — Additional file 1: Figure S1. [UMAP visualization of scRT-qPCR and scRNAseq data]. Figure S2. [PCA of scRT-qPCR and scRNAseq data]. Figure S3. [Half-life of mRNA table]. Figure S4. [Histograms of viability rate during reversion and differentiation processes]. Figure S5. [Summary table of scRNAseq data filtering steps and threshold values for each step]. [file 12915_2022_1363_MOESM1_ESM.pdf]

Supplementary

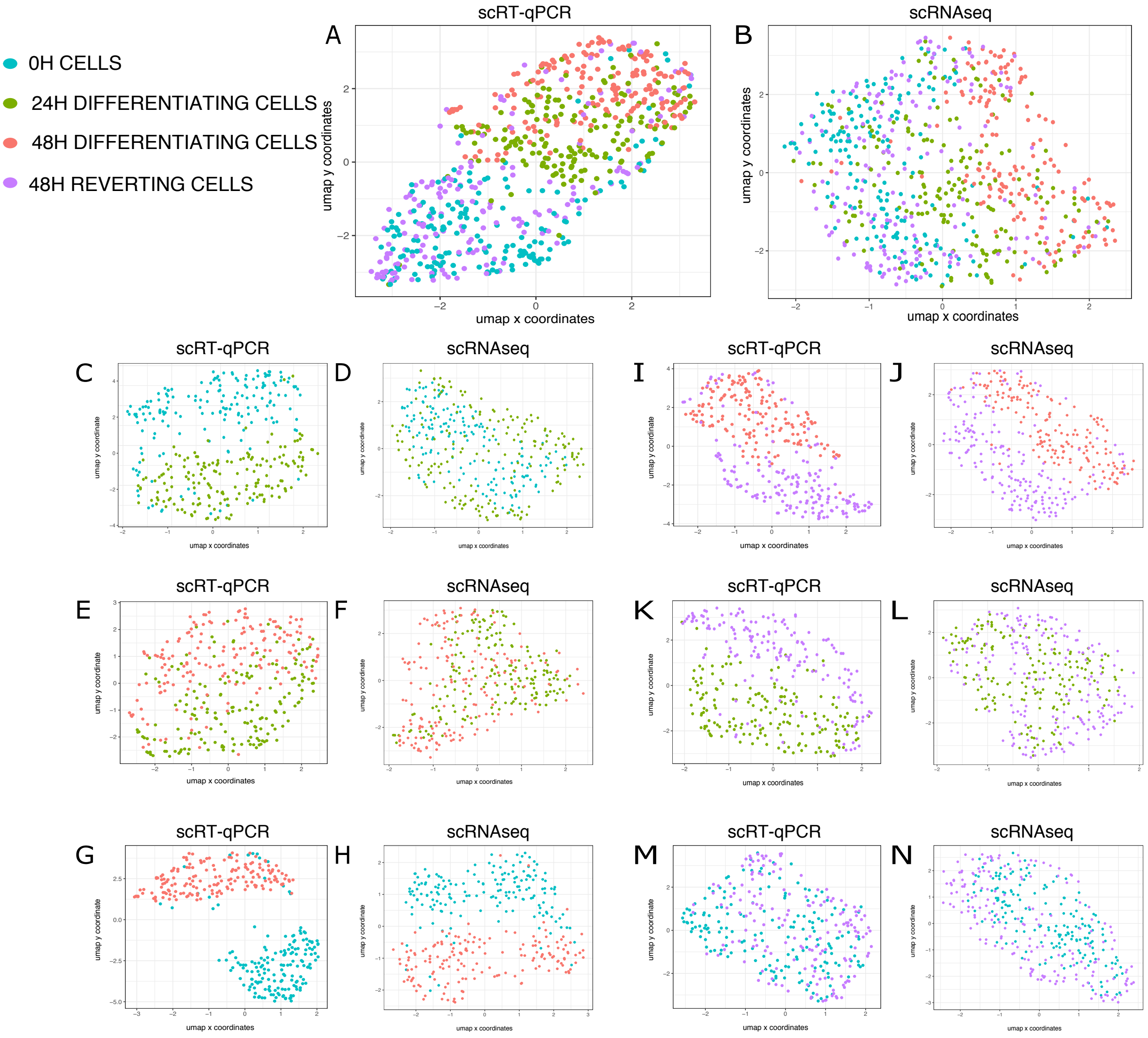

Fig. S1: UMAP visualization of scRT-qPCR and scRNAseq data. All UMAPs are recalculated when projecting conditions two by two.

Panels A, C, E, G, I, K and M: scRT-qPCR data

Panels B, D, F, H, J, L and N: scRNAseq data

Panels A and B: all 4 conditions

Panels C and D: 0H and 24H differentiating cells

Panels E and F: 24H and 48H differentiating cells

Panels G and H: 0H and 48H differentiating cells

Panels I and J: 48H differentiating and 48H reverting cells

Panels K and L: 24H and 48H reverting cells

Panels M and N: 0H and 48H reverting cells

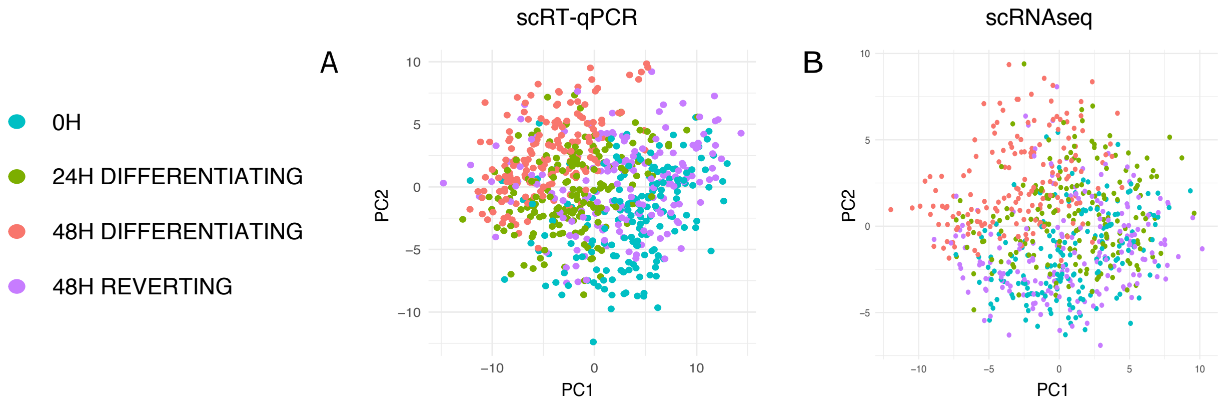

Fig. S2: Principal Component Analysis (PCA) of scRT-qPCR and scRNAseq data. PC1 and PC2 correspond to the two first components of the PCA. 0H cells are displayed in blue, 24H differentiating cells in green, 48H differentiating cells in red and 48H reverting cells in purple.

Gene Name	Ensembl_Gene_ID	mRNA half-life at 24h (hour)
HSP90AA1	ENSGALG00000011351	3,47
TBC1D7	ENSGALG00000012731	3,69
RPL22L1_1	ENSGALG00000009312	16,64
betaglobin	ENSGALG00000028273	16,64
TPP1	ENSGALG00000022706	16,64
DPP7	ENSGALG00000009105	16,70

Fig. S3: Half-life of mRNA table.

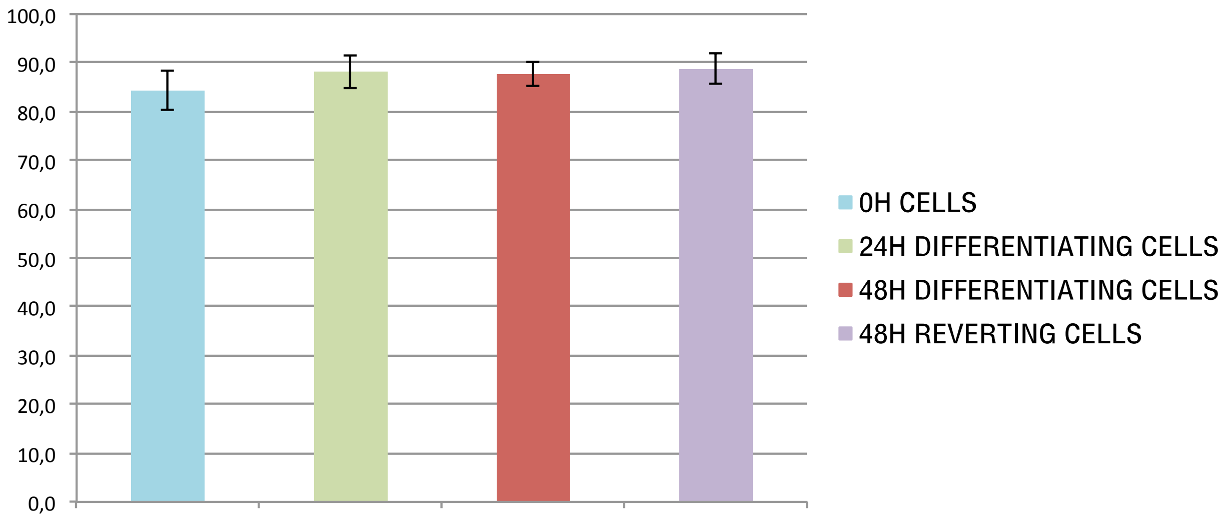

Fig. S4: Histograms of viability rate during reversion and differentiation processes measured by Trypan blue staining and counting in a Malassez chamber. Means were compared using Student t-tests.

QC step	Filter value	Number of cells remaining	Number of features remaining
**Number of sequencing reads (per cell)**	80000 reads	741 cells	/
**Percentage of mapped reads (per cell)**	46%	740 cells	/
**Percent of reads mapped to ERCC (per cell)**	<20%	710 cells	/
**Number of detected genes (per cell)**	233 genes	710 cells	/
**Number of UMIs (per cell)**	1584 UMIS	710 cells	/
**Normalization**	/	710 cells	8682 genes
**Variable features selection**	Vst 2000 most variable genes	710 cells	2000 genes

Fig. S5: Summary table of scRNAseq data filtering steps and threshold values for each step. Threshold determination is explained in methods.
